# The prevalence of comorbid migraine in multiple sclerosis: do multiple sclerosis and migraine really coexist?

**DOI:** 10.22514/jofph.2026.030

**Published:** 2026-05-12

**Authors:** Katarzyna Kępczyńska, Patryk Sochań, Natalia Bednarczyk, Patrycja J. Sochań, Wojciech Domitrz, Jan Kochanowski, Izabela Domitrz

**Affiliations:** ^1^Department of Neurology, Faculty of Medicine and Dentistry, Medical University of Warsaw, 01-809 Warsaw, Poland; ^2^Department of Neurology, Bielanski Hospital, 01-809 Warsaw, Poland; ^3^Sadyba Medical Center, 02-943 Warsaw, Poland; ^4^Faculty of Mathematics and Information Science, Warsaw University of Technology, 00-662 Warsaw, Poland

**Keywords:** Migraine, Multiple sclerosis, Comorbid migraine

## Abstract

**Background**: Multiple sclerosis (MS) is a chronic inflammatory disease causing multifocal demyelination and axonal damage in the central nervous system. Recent studies indicate that MS patients have a higher prevalence of migraine than the general population. This cross-sectional, single-centre study assessed migraine prevalence in MS patients receiving disease-modifying therapies (DMTs). **Methods**: A total of 205 MS patients were included. All participants were assessed for migraine diagnosis according to the International Classification of Headache Disorders, 3rd edition (ICHD-3), by a qualified physician. Migraine subtypes (episodic or chronic, with or without aura) were determined per ICHD-3 criteria. Each participant provided data on age, gender, current DMT type and duration, previous DMT history, MS-related symptoms, and MS relapses in the past 12 months. **Results**: Episodic migraine was identified in 36 patients, corresponding to a prevalence of 17.56% (95% CI (confidence interval): 12.4%–22.8%). Age-stratified analysis revealed higher prevalence in younger participants: 21.4% in those under 40 years (n = 98) compared to 14.0% in those aged 40 years or older (n = 107). The majority of cases (n = 28) presented without aura, with aura occurring in 8 patients. No chronic migraine was detected in the cohort. A total of 193 patients were diagnosed with relapsing-remitting multiple sclerosis (RRMS), 2 with secondary progressive MS (SPMS), and 10 with primary progressive MS (PPMS). No cases of progressive-relapsing MS (PRMS) were reported. Among the participants, 193 were receiving DMT, while 12 patients were not undergoing chronic immunotherapy. No significant correlations were found between migraine occurrence and MS type, type of DMT, disease duration, or Expanded Disability Status Scale (EDSS) score. **Conclusions**: Migraine does not seem to be less common in MS patients compared to the general population but further, age-stratified and controlled studies are needed to investigate if it is more/as common.

## 1. Introduction

Multiple sclerosis (MS) is a chronic inflammatory disease leading to multifocal neuronal demyelination and axonal damage in the central nervous system (CNS). As an autoimmune and neurodegenerative condition, MS is a leading cause of nontraumatic neurological disability in young adults, typically characterized by symptoms such as visual disturbances, spastic paresis, paresthesia, numbness, and fatigue. Although MS symptoms can vary, headaches are not considered a typical manifestation [1]. Migraine ranks as the second leading cause of disability worldwide and can be classified based on the presence or absence of aura and subdivided according to the frequency of headaches. Migraine is a headache disorder manifesting in attacks lasting 4–72 hours. Typical characteristics of migraine headache are unilateral location, pulsating quality, moderate-to-severe intensity, aggravation by routine physical activity, and association with nausea and/or photophobia and phonophobia. Individuals with chronic migraine experience headaches occurring on 15 or more days per month for longer than 3 months, with migraine features present on at least 8 of those days [2].

A recent comprehensive case-control study by Gklinos *et al*. [3] demonstrated a significant association between multiple sclerosis and both migraine and tension-type headache, providing robust evidence for increased prevalence of primary headache disorders in MS patients. A large spectrum of headache manifestations has been reported as comorbidities in MS, contributing to additional disability and adversely affecting quality of life [4, 5]. Some studies have shown that migraine commonly presents as an initial symptom in patients with MS, indicating a potential link between migraine and increased risk of developing MS [6, 7]. However, the current evidence supporting this theory is limited. It is known that migraine and MS occur within similar demographic groups and share common background factors, including gender, hormonal status, and psychological features such as anxiety, depression, and stress. In addition, some symptoms of migraine and MS may overlap, highlighting the importance of reporting the duration and nature of symptoms to a healthcare provider. While it is possible that the two conditions share some underlying mechanisms, current evidence remains limited. Over time, an increased incidence of headaches, including migraine, has been observed among patients with MS [8, 9]. The relationship between migraine and MS remains unclear. It is uncertain whether the association between headaches and MS can be attributed to shared triggers. Clinical data show that the onset of migraine typically precedes the clinical diagnosis of MS by several years. The higher prevalence of migraine in MS patients can be enhanced by disease-modifying treatments [10, 11].

Given the potential clinical significance of primary headaches in MS patients and the geographical variability in migraine prevalence, we propose that routine clinical screening of primary headaches in this population will facilitate the development of individualized treatment plans alleviating both the physical and mental burden of the disease. However, most previous studies have relied on self-report questionnaires or retrospective surveys, which may lead to symptom over-interpretation and misclassification. Therefore, the aim of this study was to assess the prevalence of migraine in MS patients treated with disease-modifying therapies (DMTs) through direct physician examination and diagnosis according to standardized criteria, and to compare these findings with population-based prevalence data from Poland.

## 2. Materials and methods

A cross-sectional, single-centre, observational study was performed to investigate the prevalence of comorbidity between migraine and multiple sclerosis (MS).

The study was conducted in accordance with all relevant institutional and national guidelines and regulations and was approved by the Bioethical Committee of the Medical University of Warsaw, Poland (reference number AK-BE/295/2024). Informed consent was obtained from all participants before enrollment.

Data collection took place over a six-month period in 2024. The participants of the study were patients enrolled in the National Health Fund’s Therapy Program for Multiple Sclerosis (MS) at the Outpatient Clinic of Bielanski Hospital, Warsaw, Poland, as well as MS patients hospitalized in the Neurology Department of the same hospital. We used convenience sampling—consecutive patients presenting to the Neurology Outpatient Clinic and hospitalized in the Neurology Department. This was not a random sample, which represents a study limitation and may affect result representativeness.

The patients diagnosed with MS prior to the study were included. Patients who declined to participate in the study were excluded. The inclusion and exclusion criteria are presented below.

Inclusion Criteria:

- Patients with confirmed diagnosis of multiple sclerosis according to McDonald 2017 criteria;

- Age ≥18 years;

- Ability to provide informed consent for study participation;

- Patients receiving disease-modifying therapies (DMT), including interferon beta, glatiramer acetate, fingolimod, dimethyl fumarate, teriflunomide, natalizumab, alemtuzumab, ocrelizumab, as well as treatment-naive patients.

Exclusion Criteria:

- Refusal to participate in the study;

- Secondary headaches related to other neurological conditions (other than MS);

- Medication overuse headaches;

- Active central nervous system infections;

- Severe cognitive impairment preventing interview conduct;

- Headaches related to head trauma within the last 3 months.

All participants were interviewed and examined by physicians experienced in diagnosing and treating patients with headaches, under the supervision of a neurologist specializing in headaches.

Every patient (including those who reported experiencing migraine or migraine-like headaches) was diagnosed for migraine according to the International Classification of Headache Disorders, 3rd Edition (ICHD-3), by a physician [2]. Patients diagnosed with migraine were subsequently assessed to determine the subtype (episodic or chronic, with or without aura) according to the ICHD-3 criteria.

Migraine diagnosis was established based on detailed medical history conducted by experienced neurologists. Patients were asked about headache occurrence during the last 12 months, with particular attention to characteristics, frequency, intensity, and accompanying symptoms according to ICHD-3 criteria. For each headache episode, we assessed: location, pain character, intensity (1–10 scale), duration, triggering and alleviating factors, and presence of accompanying symptoms (nausea, vomiting, photophobia, phonophobia). An important limitation of our study is the lack of prospective headache diary use, which could have increased the accuracy of migraine diagnosis and reduced recall bias. Future studies should incorporate prospective headache monitoring using standardized diaries.

Every participant was directly asked by a physician about their gender, age, place of living (urban or rural), marital status, parental status, education level, employment status, and comorbidities. Additional information included the age at onset of MS symptoms, age at MS diagnosis, type of first symptoms (motor, sensory, optic, cerebellar, brainstem, spinal cord or multifocal), and the number of MS relapses in the past 12 months. Moreover, participants were asked about diagnostic tests performed anytime in their medical history, including brain magnetic resonance imaging, cerebrospinal fluid analysis, optical coherence tomography, and visual evoked potentials. Every patient was asked about MS disease-modifying therapy (type, duration of therapy, previous disease-modifying therapies). A neurological examination was performed for each participant, and Expanded Disability Status Scale (EDSS) score was assessed.

Descriptive statistics were used to characterize the study population. Continuous variables are presented as means ± standard deviations (SD) and categorical variables as frequencies and percentages. A 95% confidence interval (CI) for the prevalence of migraine in the MS population was calculated. Statistical analyses were performed using Statistica (version 13.0, TIBCO Software Inc., Palo Alto, CA, USA). A *p*-value < 0.05 was considered statistically significant.

## 3. Results

A confidence interval for the proportion of migraine in MS population was 95% CI: 12.4%–22.8%. A total of 205 MS patients were included in the study (149 women and 56 men), with a mean age of 39.87 ± SD 10.65 years (range: 18 to 72 years) (Fig. 1). Among them, 171 patients lived in urban areas, while 34 resided in rural areas. Detailed information about MS relapses in the past year was collected during patient interviews. All characteristics of the study population are presented in Table 1.

**Fig. 1.** Age Distribution of Study Participants. The histogram shows the age distribution of 205 MS patients, with particular attention to the 18–39 years age group (n = 98) and 40 years or older age group (n = 107), which is relevant for migraine epidemiology as migraine prevalence peaks in the third and fourth decades of life.

**Table 1.** Characteristics of the study population.

Characteristic		Value
Age, yr (mean ± SD)		39.87 ± 10.65
Gender, n (%)		
	Female	149 (72.7%)
	Male	56 (27.3%)
Education		
	Primary	2 (1.0%)
	Vocational	13 (6.3%)
	Secondary	49 (24.0%)
	Higher	141 (68.8%)
Residence, n (%)		
	Urban	171 (83.4%)
	Rural	34 (16.6%)
MS phenotype, n (%)		
	RRMS	193 (94.1%)
	SPMS	2 (1.0%)
	PPMS	10 (4.9%)
MS duration, yr (mean ± SD)		7.72 ± 7.22
EDSS score (mean ± SD)		2.26 ± 1.27
DMT use, n (%)		
	Yes	193 (94.1%)
	No	12 (5.9%)
Relapses (last 12 mon), n (%)		
	0	152 (74.1%)
	1	48 (23.4%)
	2	5 (2.4%)

*RRMS, relapsing-remitting multiple sclerosis; SPMS, secondary progressive multiple sclerosis; PPMS, primary progressive multiple sclerosis; DMT, disease-modifying therapy; EDSS, Expanded Disability Status Scale; SD, standard deviation; MS, Multiple sclerosis.*

Thirty-six patients (17.56%, 95% CI: 12.4%–22.8%) were diagnosed with episodic migraine. Among patients aged <40 years (n = 98), 21 (21.4%) had migraine, compared to 15 patients (14.0%) among those aged ≥40 years (n = 107). Among them, 28 patients had migraine without aura (13.66% of all the participants, 77.78% of the patients with migraine), while 8 patients had migraine with aura (3.90% of all the participants, 22.22% of the patients with migraine). Notably, no patient in the study was diagnosed with chronic migraine. According to the current MS phenotype classification, all patients were classified as: RRMS (n = 193), SPMS (n = 2), or PPMS (n = 10). Migraine characteristics in the study population are presented on Table 2.

**Table 2.** Migraine characteristics in the study population.

Migraine subtype	n (%)
Total migraine	36 (17.56)
Episodic migraine	36 (100.0)
Chronic migraine	0 (0.0)
Migraine without aura	28 (77.8)
Migraine with aura	8 (22.2)

A total of 193 participants were receiving disease modifying therapy (DMT), while 12 patients remained without chronic immunotherapy. The mean MS duration was 7.72 ± 7.22 years. The mean EDSS score was 2.26 (range: 1 to 6.5).

We did not find any correlation between the type of MS, the type of DMTs, the duration of MS, or EDSS score and migraine occurrence.

The medical history of all patients included assessment of relapses within the year prior to the study. A total of 152 patients had no relapses, 48 patients had 1 relapse, 5 participants had 2 relapses. No participants had more than 2 relapses during this period. The high percentage of patients without relapses in the past year (74.1%) may reflect the effectiveness of modern disease-modifying therapies and the fact that most patients were recruited during routine outpatient visits rather than during hospitalizations for relapses.

Moreover, 29 patients reported a family history of MS, while 176 participants did not report any familial diagnosis of the condition. No significant evidence was found supporting a link between the number of relapses, the presence of migraine, or a family history of MS.

To exclude other pathologies that could affect the prevalence of migraine in MS patients, we analyzed the occurrence of additional comorbidities. Among the 205 participants, 116 patients (56.6%) had no comorbid conditions, while 89 (43.4%) reported at least one. Cardiovascular comorbidities (including hypertension and heart disease) were present in 35 patients (17.1%). Specifically, hypertension was noted in 29 patients (14.15%) and heart disease in 6 patients (2.93%). The most frequent comorbidities were hypertension (29/205; 14.15%), thyroid diseases (28/205; 13.66%), and depression (22/205; 10.73%). Less common conditions included diabetes mellitus (6/205; 2.93%) and heart disease (6/205; 2.93%). Additionally, 34 patients (16.59%) reported other comorbid conditions (Fig. 2). No significant correlation between migraine and the presence of comorbidities was found.

**Fig. 2.** Concomitant diseases among study participants.

## 4. Discussion

Some literature data suggest that migraine-type headaches occur more frequently in MS patients than in the general population [4, 5, 6]. Based on these findings, we estimated the comorbidity of migraine and MS in a Polish population. Our patients where directly interviewed and examined by physicians. The obtained results suggest that there is no strong evidence that MS is associated with migraine. Only 36 (17.6%) out of 205 MS patients were diagnosed with migraine. The limited number of patients with progressive MS forms (n = 12) does not allow for drawing definitive conclusions regarding differences in migraine frequency between different disease phenotypes. A thorough medical history and detailed neurological examination were necessary to rule out secondary headache syndromes. Our observations align with the reported prevalence of migraine in the general population. This is in line with the study of Ashina *et al*. [11], which reported that migraine affects over 1 billion people worldwide, with particularly high prevalence rates. Based on the results from the 2021 Global Burden of Disease (GBD) study, 18% of people both in Poland and globally, aged 20 and older, experience migraine [12, 13]. Our findings of 17.56% migraine prevalence in MS patients closely align with this Polish population estimate. However, we acknowledge that GBD estimates may have limitations depending on the availability of country-specific primary data sources, and ideally, comparison should be made with Polish population-based epidemiological studies when available.

On the other hand, some studies report a lower prevalence of migraine: 14.0% globally and 16.6% in Central Europe [14]. Migraine and MS affect similar demographic groups that share common background, including gender. These conditions are more prevalent in women and particularly in younger individuals (in the third and fourth decades of life) [15]. The mean age of our patients was 39.87 years, with a higher proportion of women than men (149 women *vs.* 56 men). These factors may contribute to the occurrence of migraine, but the relationship between migraine and MS remains unclear. Further studies are needed to confirm any potential associations between these two diseases. Age stratification of our results revealed interesting patterns. Among patients aged <40 years, migraine prevalence was 21.4%, while in patients ≥40 years it was 14.0%. Given the natural course of migraine, which typically declines with age, the lower prevalence in the older age group likely reflects underascertainment due to migraine remission rather than true absence of lifetime migraine history. Therefore, the <40 age group prevalence of 21.4% is more representative of the cumulative incidence and lifetime prevalence of migraine in our MS population and should be emphasized in comparisons with general population data. The overall prevalence of 17.56% in our mixed-age population reflects the demographic composition of our sample, with a mean age of 39.87 years. Future should consider age-specific prevalence rates when comparing MS populations to general population data.

One of the studies based on a meta-analysis suggests that the prevalence of headache disorders can be higher in MS patients. However, we should underline that most of the studies included in this meta-analysis were of low-to-moderate quality due to methodological differences [10]. Moreover, Mirmosayyeb *et al*. [15] reported that the prevalence of migraine can differ in each continent, with pooled estimates of 24% in Asia, 43% in Americas, 25% in Europe, and 43% in Africa. In line with this observation, Fragoso *et al*. [16] conducted a study in which MS patients, who also experienced headaches, were invited to complete an online survey. Migraine was identified in 54.1% of patients, with 68.3% reporting a moderate-to-high migraine burden. In many studies reporting a higher prevalence of migraine in a group of MS patients compared to the general population, data were collected using questionnaires. A meta-analysis performed by Gklinos *et al*. [10] found that migraine is more common in MS patients than in the general population. However, in only 5 out of 23 studies included in the meta-analysis, migraine diagnosis was confirmed through a medical interview conducted by a physician. In relation to these observations, our study ensured that MS patients were directly diagnosed and examined by physicians experienced in headache disorders. They did not complete the survey themselves, which increased the accuracy of migraine diagnosis.

Self-diagnosis can often lead to misclassification, with many patients confusing migraine with tension-type or migraine-type headache. Moreover, during the interviews, many patients initially reported experiencing photophobia, but upon more detailed history taking, they specified that they simply “did not like looking at light”, which was unrelated to any headache episodes. Similar misunderstandings are more likely to occur when using questionnaires.

A particularly interesting observation was performed by Gklinos *et al*. [17] The authors were trying to determine if the prevalence of migraine-like headaches in MS patients is associated with plaques in the brainstem or lesions in other brain regions. Recent evidence, including the comprehensive cross-sectional study by Gklinos *et al*. [18], has demonstrated a strong association between periaqueductal gray (PAG) lesions and migraine in MS patients, providing robust support for anatomical correlates of headache in this population. This finding was also presented at the recent European Neurological Congress in Helsinki, highlighting the growing recognition of this important association. According to ICHD-3, migraine can be diagnosed when other causes of headache have been excluded. Patients with brainstem plaques may experience secondary headaches and be misdiagnosed with migraine. This observation requires further research.

Özer *et al*. [19] reported that headache may be the only symptom of a flare-up in MS patients, with headache and MS relapse occurring concurrently in 23.6% of RRMS patients. It is important to consider if these headaches can be classified as migraines, which are primary headaches. Comprehensive assessment of all primary headache types could provide a more complete picture of the relationship between MS and headache disorders. Headaches occurring during MS relapses should be classified as secondary headaches associated with the underlying disease.

The medical history of our patients included relapses at least once in the year prior to the study enrollment. However, we found no correlation between relapses and migraine. The reason for that may be that the vast majority of participants were interviewed and examined during outpatient clinic visits, and experienced no relapses during that time. Hence, it was not possible to assess headache symptoms during relapse episodes directly.

To sum up, our results indicating comparable migraine prevalence in MS patients and the general population require detailed interpretation in the context of previous studies suggesting higher prevalence. Maybe methodological differences between studies may significantly influence obtained results. Many previous studies relied on self-report questionnaires or retrospective surveys, which may lead to symptom over-interpretation and misclassification of tension-type headaches as migraine. In our study, each patient was directly examined by experienced neurologists, which may have increased diagnostic precision. Moreover, modern disease-modifying therapies for MS may influence headache frequency and severity. The majority of our patients (94.1%) were receiving DMT, which could have affected migraine frequency reduction through inflammatory process control and disease course stabilization. It is also possible that previous studies included patients with more active MS forms or at different disease stages, while our group was characterized by relatively stable disease course (74.1% without relapses in the last year). Our study population was characterized by relatively stable disease course, with 74.1% of patients experiencing no relapses in the past year and 94.1% receiving DMTs. This predominantly stable patient population may have influenced our results in several ways. Modern DMTs may influence headache frequency through inflammatory process control and disease course stabilization. Additionally, patients with more active MS or during relapse periods may have different headache patterns. Most patients were recruited during routine outpatient visits rather than during hospitalizations for relapses, which may have affected the observed migraine prevalence. Finally, cultural, genetic, and environmental differences between populations may influence migraine prevalence, which may explain discrepancies between studies conducted in different geographical regions.

## 5. Study limitations

Our study did not include a control group of healthy individuals matched by age, sex, and sociodemographic factors. While we compared our results to well-established general population prevalence data from the Global Burden of Disease Study and other epidemiological studies, direct comparison with an examined control group would have strengthened our conclusions. However, comparison with established population prevalence data is a valid epidemiological approach widely used in prevalence studies, particularly when population-level estimates are robust and well-documented.

Our study lacked statistical power for robust association analyses, particularly for rare comorbidities. No multivariable analysis was performed to adjust for potential confounders. Therefore, negative findings regarding associations between migraine and MS characteristics should be interpreted with caution.

Our study did not include detailed MRI (Magnetic Resonance Imaging) analysis in the context of demyelinating lesion location and burden. Future studies should investigate the potential relationship between specific MRI lesion locations (particularly in the periaqueductal gray area, brainstem, and other structures related to pain processing) and migraine occurrence and severity in MS patients.

Our study has also several important limitations that should be considered when interpreting the results. First, this is a cross-sectional study with retrospective headache assessment, which introduces significant recall bias, particularly in MS patients who may experience cognitive impairment related to their disease. The retrospective design also does not allow for establishing robust causal relationships between MS and migraine.

Second, we did not utilize prospective headache diaries for migraine diagnosis, which could have increased diagnostic accuracy and reduced assessment subjectivity. Future studies should incorporate prospective symptom monitoring using standardized tools.

This is a single-center study with convenience sampling of consecutive patients, which may introduce selection bias and limit the generalizability of results to the broader MS patient population. The predominantly stable patient population (74.1% without relapses in past year, 94.1% receiving DMTs) may not be representative of all MS phenotypes or disease activity states. Differences in clinical practice, population characteristics, and healthcare accessibility may influence the obtained results.

Finally, our study did not incorporate standardized migraine severity assessment scales (such as MIDAS—Migraine Disability Assessment Scale) or their correlation with MS clinical parameters such as EDSS, disease duration, or relapse frequency. Future studies should include comprehensive assessment of migraine’s impact on quality of life and its relationship with MS activity and progression.

## 6. Conclusions

Migraine is a common disease both in the general population and in MS patients. Our study focused exclusively on migraine, not considering other primary headaches such as tension-type headache. Future studies should encompass a broader spectrum of primary headaches, as patients may experience different headache types throughout their lifetime, and their coexistence may complicate accurate diagnosis. According to our results, migraine does not appear to be less common in MS patients compared to the general population. However, further age-stratified and controlled studies are needed to investigate whether migraine is equally or more common in MS patients, and to explore potential differences across MS phenotypes and disease activity states. However, further research involving larger sample sizes and the inclusion of control groups is necessary to confirm this association.
